# Comparative genomic sequencing to characterize *Mycoplasma pneumoniae* genome, typing, and drug resistance

**DOI:** 10.1128/spectrum.03615-23

**Published:** 2024-06-21

**Authors:** Yue Jiang, Hailong Kang, Haiwei Dou, Dongxing Guo, Qing Yuan, Lili Dong, Zhenglin Du, Wenming Zhao, Deli Xin

**Affiliations:** 1 Pediatric Department, Beijing Chao-Yang Hospital, Capital Medical University, Beijing, China; 2 National Genomics Data Center and CAS Key Laboratory of Genome Sciences and Information, Beijing Institute of Genomics, Chinese Academy of Sciences and China National Center for Bioinformation, Beijing, China; 3 College of Life Sciences, University of Chinese Academy of Sciences, Beijing, China; 4 Tropical Medicine Research Institute, Beijing Friendship Hospital, Capital Medical University, Beijing, China; 5 School of Future Technology, University of Chinese Academy of Sciences, Beijing, China; Southern Medical University, Guangzhou, China

**Keywords:** *Mycoplasma pneumoniae*, macrolide resistance, comparative genomics, P1 genotype, clinical manifestation

## Abstract

To analyze the characteristics of *Mycoplasma pneumoniae* as well as macrolide antibiotic resistance through whole-genome sequencing and comparative genomics. Thirteen clinical strains isolated from 2003 to 2019 were selected, 10 of which were resistant to erythromycin (MIC >64 µg/mL), including 8 P1-type I and 2 P1-type II. Three were sensitive (<1 µg/mL) and P1-type II. One resistant strain had an A→G point mutation at position 2064 in region V of the 23S rRNA, the others had it at position 2063, while the three sensitive strains had no mutation here. Genome assembly and comparative genome analysis revealed a high level of genome consistency within the P1 type, and the primary differences in genome sequences concentrated in the region encoding the P1 protein. In P1-type II strains, three specific gene mutations were identified: C162A and A430G in L4 gene and T1112G mutation in the CARDS gene. Clinical information showed seven cases were diagnosed with severe pneumonia, all of which were infected with drug-resistant strains. Notably, BS610A4 and CYM219A1 exhibited a gene multi-copy phenomenon and shared a conserved functional domain with the DUF31 protein family. Clinically, the patients had severe refractory pneumonia, with pleural effusion, necessitating treatment with glucocorticoids and bronchoalveolar lavage. The primary variations between strains occur among different P1-types, while there is a high level of genomic consistency within P1-types. Three mutation loci associated with specific types were identified, and no specific genetic alterations directly related to clinical presentation were observed.

**IMPORTANCE:**

*Mycoplasma pneumoniae* is an important pathogen of community-acquired pneumonia, and macrolide resistance brings difficulties to clinical treatment. We analyzed the characteristics of *M. pneumoniae* as well as macrolide antibiotic resistance through whole-genome sequencing and comparative genomics. The work addressed primary variations between strains that occur among different P1-types, while there is a high level of genomic consistency within P1-types. In P1-type II strains, three specific gene mutations were identified: C162A and A430G in L4 gene and T1112G mutation in the CARDS gene. All the strains isolated from severe pneumonia cases were drug-resistant, two of which exhibited a gene multi-copy phenomenon, sharing a conserved functional domain with the DUF31 protein family. Three mutation loci associated with specific types were identified, and no specific genetic alterations directly related to clinical presentation were observed.

## INTRODUCTION

*Mycoplasma pneumoniae (M. pneumoniae*) is an important pathogen of community-acquired pneumonia (CAP) in children, accounting for approximately 10%–40% of CAP in children and more common in older children, accounting for 20% of CAP in adults. Infection rates tend to be higher in specific populations with close contact, such as schools and military settings. The epidemic period typically lasts for about 3–4 years (1–3). While most *M. pneumoniae* infections are self-limiting, in some cases, it can lead to pneumonia or combined multiple complications both within and outside the lungs. These complications may include pulmonary solidus, pleural effusion, rash, and Steven-Johnson Syndrome, resulting in severe or refractory pneumonia and posing a potential threat to life (4).

Macrolide antibiotics are commonly used as the first-line treatment for *M. pneumoniae* infections in children. However, since 2000, the emergence of clinical macrolide-resistant strains has been reported worldwide, with different resistance rates and a higher rate in East Asia. Macrolide antibiotic resistance primarily involves three mechanisms: target modification, efflux mechanism, and antibiotic degradation (5). In *M. pneumoniae*, the primary resistance mechanism is the target mutation at site 2063 A→G in the 23S rRNA V region, which leads to high drug resistance, and no other macrolide-resistant mechanisms have been reported. Considering that children are in a critical phase of growth and development, the selection of antibiotics is often limited. The inability to promptly change effective antibiotics may be associated with increased clinical severity and refractory cases (6, 7). However, the pathogenic mechanisms of *M. pneumoniae* are complex and involve various factors, including direct cellular damage, tissue damage, and immune dysregulation (8). Our current understanding of these mechanisms remains limited.

The *M. pneumoniae* genome is a double-stranded circular DNA with a size of approximately 820 kb and over 600 protein-coding genes, indicating a small and simple structure. Due to its highly streamlined genome, *M. pneumoniae* cannot synthesize essential molecules such as amino acids and nucleotides from scratch. Instead, it relies on close binding to host cells to take up essential substances such as cholesterol, and the *in vitro* culture conditions for *M. pneumoniae* are also demanding (1). Since the release of genome information of the first *M. pneumoniae* strain in 1996, our understanding of the organism’s biological properties has significantly enhanced at the genetic level. Based on the repetitive sequences of the P1 protein-coding genes Rep2/3 and Rep4, which are part of the apical adhesion structure, *M. pneumoniae* can be simply classified into P1-type I and P1-type II, with a high degree of genomic conservation within each type. Epidemiological surveillance of *M. pneumoniae* showed that P1-type I was the predominant strain with a high percentage of drug resistance, whereas, since 2015, there has been a gradual increase in P1-type II in multiple countries (9, 10).

Genomics provides vital information for studying the structure, composition, function, and evolution of *M. pneumoniae* on a genome-wide scale. With advancements in molecular biology and bioinformatics, genomic analysis of *M. pneumoniae* helps us understand its epidemiology, drug resistance, and evolutionary characteristics, thereby offering guidance for clinical practice (1, 11). However, there is a scarcity of studies that integrate genomic information of *M. pneumoniae* with clinical manifestations. In this study, clinical isolates of *M. pneumoniae* from different generations were selected for whole-genomic sequencing, drug resistance detection, and P1 typing. The clinical manifestations were analyzed to explore new mechanisms.

## MATERIALS AND METHODS

### Clinical information collection and sample purification

A total of 13 strains of culture-positive pharyngeal swab samples from 2003 to 2019, in Beijing, China, were selected separately, and the children were diagnosed according to guidelines [Guidelines for the management of community-acquired pneumonia in children (the revised edition of 2013) (I)] (12) for the diagnosis of *M. pneumoniae* pneumonia (MPP). Severe *M. pneumoniae* pneumonia (SMPP) is diagnosed if there are signs of severe pneumonia such as ultra-high fever or persistent high fever, dyspnea, lung lesions involving more than two-third of the lung tissue, and combined intra-pulmonary and extra-pulmonary complications. Among them, regular treatment with macrolide antibiotics for ≥7 days with persistent fever and no improvement or worsening of pulmonary imaging was considered refractory *M. pneumoniae* pneumonia (RMPP), which mostly presented as a severe disease. Permission was obtained from the child or guardian for the collection of clinical information and samples. All the strains were isolated and stored in the laboratory of Tropical Medicine Research Institute, Beijing Friendship Hospital, Capital Medical University, Beijing, China.

### Sample purification, drug susceptibility testing, and genomic DNA preparation

Take 0.1 mL of preserved bacterial solution and inoculate it in a pleuropneumonia-like organism (PPLO) solid medium. Observe daily until fried egg-like mature colonies form. Pick a single clone and transfer it to a PPLO liquid medium. Growth is indicated by a color change from red to yellow. Identify the species as *M. pneumoniae* using 16S rRNA. Purified samples are used for erythromycin *in vitro* susceptibility testing, with erythromycin standards purchased from China. The MIC value of erythromycin was measured by microdilution method, and the strain was considered sensitive if it was ≤0.5 µg/mL and resistant if it was ≥1 µg/mL. The same process was performed for FH and M129 during the experiment as a reference. The identified *M. pneumoniae* strains were cultured in increments, extracted and purified using a DNA extraction kit (Kangwei Reagent “Universal Column Genome Extraction Kit,” item number CW2298), and stored at −80°C until use. At the same time, FH (ATCC15531) and M129 standard strains [both purchased from ATCC (American Type Culture Collection)] kept in the laboratory were cultured and performed DNA extracted, then stored at −80°C until use.

### P1 typing and drug resistance gene detection

P1 typing was performed according to the Rep2/3 and Rep4 repeat sequences of P1 protein-coding genes with reference to previous literature (13). Primers to amplify region II and region V of 23S rRNA were self-designed primers (Table S1).

### Public data download

The genome sequence (accession number NZ_CP017343.1) and annotation information of the P1-type I reference strain M129, along with the raw sequencing data (accession number SRR3924617), and the genome sequence (accession number NZ_CP017327.1) and annotation information of the reference strain FH, which belongs to P1-type II, were downloaded from the NCBI website.

### Genome assembly

#### Next-generation sequencing and assembly

The genomic DNA of 11 clinical isolates of *M. pneumoniae* was sequenced using the Illumina HiSeq 2000 platform at the Center for Innovation in Genome Technology, Beijing Institute of Genomics, Chinese Academy of Sciences. The paired-end sequencing had a length of 100 bp, and the average sequencing depth was 1,435× (ranging from 871× to 1,702×). Quality control for raw sequencing data was performed using FastQC software (https://www.bioinformatics.babraham.ac.uk/projects/fastqc/). The SOAPdenovo2 software (14) was used for *de novo* assembly by iteratively enumerating possible kmer values from 13 to 63. The assembly results performed superior performance with k-mer values of 43, 45, and 47. Among these values, we selected the assembly with the highest N50 value. The obtained assembly was subsequently mapped against the reference genome sequence.

#### Third-generation sequencing and assembly

Due to the unavailability of samples for strains 604b, 828B, and M30B, only 8 out of the initial 11 strains underwent third-generation DNA sequencing library construction. Additionally, to ensure a balanced representation of drug-resistant and sensitive strains, we included two additional strains, BS610A4 and CYM219A1, for third-generation sequencing. Strain 16c was sequenced using the Pacific Biosciences RS II sequencing platform at the Center for Innovation in Genome Technology, Beijing Institute of Genomics, Chinese Academy of Sciences. The sequencing data were assembled using Falcon software, followed by correction using NextPolish software. The remaining nine strains, excluding 16c, were sequenced using the Pacific Biosciences Sequel I sequencing platform at Annaroad Gene Technology (Beijing) Co., Ltd. The resulting third-generation sequencing data were then assembled using Falcon software, followed by correction using Unzip software. The assembled results were further evaluated for quality using the QUAST software (15) and BUSCO software (16).

### Genome annotation

The *de novo* assembly results obtained from both second- and third-generation sequencing data were annotated using PGAP software (17).

### SNP and InDel analyses

Single nucleotide polymorphism (SNP) and insertion-deletion (InDel) analyses were performed on all clinical isolates. The clean reads were aligned to the M129 reference genome (NCBI accession number NZ_CP017343.1) using the BWA software (18). The resulting BAM files were used as input for the HaplotypeCaller module from the GATK software (19) to identify SNP and InDel. For strains that only had third-generation whole-genome sequencing data, SNP and InDel analysis was conducted using the MUMmer software (20) based on the genome sequence. The functional annotation of the variants in the resulting VCF files was performed using the SnpEff software (21), and the distribution of genomic variations across the strains was visualized using the R package ComplexHeatmap (22).

### Evolutionary tree construction

Based on all SNP loci, a fasta file was generated containing sequences of equal length for each strain. This fasta file was then used as input for multiple sequence alignment using the Clustal W software (23). After the alignment process, an evolutionary tree was constructed using the MEGA X software (24), employing the maximum likelihood method with 1,000 iterations of bootstrap analysis to ensure the generation of a high-confidence tree structure.

### Comparative genomic analysis

The completed genomes were compared using BRIG software (25) to determine the genome sequence similarity between the strains. Additionally, the Roary software (26) was utilized to identify differential genes within each strain by comparing the genome annotation files.

### Screening of drug-resistance genes

The nucleotide sequences of relevant macrolide resistance genes were downloaded from the Comprehensive Antimicrobial Resistance Database (CARD) (27) and NCBI. These resistance gene sequences were aligned to the genomic sequences of each strain using the BLAST+ software (28). Subsequently, the presence or absence of the resistance gene was determined based on the alignment results, applying the following thresholds: (i) alignment sequence length >80% in each sequence and (ii) sequence identity >80%.

## RESULTS

### Clinical information

All 13 children, aged 3–13 years, were diagnosed with MPP. Among them, there were 6 males and 7 females. A total of seven cases were classified as SMPP with associated complications such as pleural effusion, abnormal liver function, and myocardial damage. All severe cases received glucocorticoid treatment, and three of them also underwent bronchoalveolar lavage (Table 1).

**TABLE 1:** Information of the clinical isolations^*a*^

Strain	Year	Age	Gender	Type of MPP	Complications	P1 typing	MR mutations	Erythromycin MIC* (µg/mL）	MR
16c	2003	6	F	SMPP	Liver dysfunction and pleural effusion	I	A2064G	>64	R
YYM3A	2004	10	F	MPP	No complication	II	–	0.06	S
M30B	2004	5	M	MPP	No complication	II	–	0.06	S
120c	2005	6	F	MPP	No complication	II	–	0.06	S
317B	2006	8	M	SMPP	Myocardial damage and metabolic acidosis	I	A2063G	>64	R
604b	2008	3	F	MPP	No complication	I	A2063G	>64	R
794B	2009	7	M	MPP	No complication	I	A2063G	>64	R
828B	2009	6	M	MPP	No complication	I	A2063G	>64	R
1037C	2010	4	M	SMPP	Liver dysfunction and myocardial damage	I	A2063G	>64	R
1063A6	2010	13	F	SMPP	Myocardial damage	I	A2063G	>64	R
CYM219A1	2016	10	F	SMPP/RMPP	Pleural effusion	II	A2063G	>64	R
BS362A2B1	2017	9	M	SMPP/RMPP	Liver dysfunction and pleural effusion	I	A2063G	>64	R
BS610A4	2019	7	F	SMPP/RMPP	Liver dysfunction, pleural effusion, and myocardial damage	II	A2063G	>64	R

^*a*^ MIC = minimum bacteriostatic concentration, S = drug-sensitive strain, erythromycin MIC ≤ 0.5 μg/mL; R = drug-resistant strain, erythromycin MIC ≥ 1 μg/mL. A dash (–) indicates no mutations.

### P1 typing and drug sensitivity testing

The 13 samples were classified into eight P1-type I strains and eight P1-type II strains based on P1 genotyping. Furthermore, the erythromycin drug sensitivity test categorized them into eight drug-resistant strains and five sensitive strains. Among the drug-resistant strains, 16C exhibited an A2064G point mutation, while the remaining strains had an A2063G point mutation. No other mutations were detected, and none of the sensitive strains showed any mutations (Table 1). In cases infected with P1-type I strains, five out of eight were classified as severe, while among cases infected with P1-type II strains, two out of five were classified as severe. Notably, all severe cases were infected with drug-resistant strains, and there was no statistically significant difference in the rate of severe cases between the two P1 types (*P* = 0.898).

### Genome assembly

The whole-genome sequencing and *de novo* assembly were performed on 13 strains, and the genome of each strain was annotated. The specific assembly results and genome composition are presented in Table 2. The strains sequenced with third-generation sequencing data yielded complete genome sequences, with a consistent number of annotated genes across different strains. However, strains that were solely sequenced with next-generation sequencing data could not form a complete genome sequence due to limitations in read length, resulting in variations in the number of annotated genes among the strains.

**TABLE 2:** The genome annotation statistics and variants^*a*^

P1-type	Strain	MR	The genome annotation statistics									Variants relative to the M129 reference strain									
			Assay type	Length	N50	Genes						Missense	Synonymous	Noncoding	Start	Stop	Splice	Inframe	Frameshift	Unknown	Total
						CDS	rRNA	tRNA	ncRNA	Pseudo gene	Total										
Type I	1037C	R	sgs + tgs	816,581	816,581	725	3	37	3	40	768	145	69	1	1	4	0	7	18	23	268
	16c	R	sgs + tgs	816,325	816,325	723	3	37	3	43	766	130	64	1	1	4	0	4	22	27	253
	317B	R	sgs + tgs	816,502	816,502	724	3	37	3	42	767	147	71	1	1	3	0	8	22	25	278
	604b	R	sgs	762,940	47,902	728	6	34	3	78	771	126	72	1	1	2	0	8	21	33	264
	794B	R	sgs + tgs	816,533	816,533	727	3	37	3	41	770	141	66	1	1	3	0	7	18	23	260
	828B	R	sgs	798,367	89,971	767	7	38	3	58	815	138	66	1	1	3	0	6	18	24	257
	BS362A2B1	R	sgs + tgs	816,564	816,564	725	3	37	3	42	768	163	68	1	1	4	0	7	28	26	298
	1063A6	S	sgs + tgs	816,630	816,630	725	3	37	3	41	768	142	68	0	1	4	0	7	18	23	263
Type II	120C	S	sgs + tgs	817,128	817,128	726	3	36	3	51	768	623	392	6	0	17	1	16	57	163	1,275
	M30B	S	sgs	815,427	231,658	793	6	36	3	65	838	625	420	7	0	18	1	16	51	158	1,296
	YYM3A	S	sgs + tgs	817,210	817,210	727	3	36	3	49	769	626	394	6	0	17	1	15	57	164	1,280
	FH	S	–	–	–	–	–	–	–	–	–	616	401	5	0	17	1	18	56	162	1,276
	CYM219A1	R	tgs	818,580	818,580	730	3	36	3	46	772	659	468	7	0	22	1	18	56	157	1,388
	BS610A4	R	tgs	818,816	818,816	729	3	36	3	48	771	663	465	7	0	21	1	19	58	155	1,389

^*a*^ sgs: next-generation sequencing; tgs: third-generation sequencing; CDS: coding sequence; rRNA: ribosomal RNA; tRNA: transfer RNA; non-coding RNA: other non-coding RNAs excluding rRNA and tRNA; Start: start_lost variant; Stop: stop_gained variant, stop_lost variant, and stop_retained variant; Splice: splice_region variant. The FH strain is solely utilized for variation analysis and does not contain assembly data, as indicated by dashes.

Strains 604b, 828B, and M30B were exclusively sequenced using next-generation sequencing. For these three strains, we merged multiple contigs to generate complete genomes by aligning them with the reference genome. To validate the accuracy of the correction results, we compared the corrected sequences with the reference genome using the MUMmer software. The alignment results exhibited a prominent diagonal distribution pattern (Fig. S1), confirming the overall accuracy of our corrections. The total length of the corrected sequences closely corresponded to the size of the reference genome (820 kb; Table S2), and the genetic integrity within the genome was significantly enhanced (Fig. S2).

### Genome comparison

**Fig 1:**
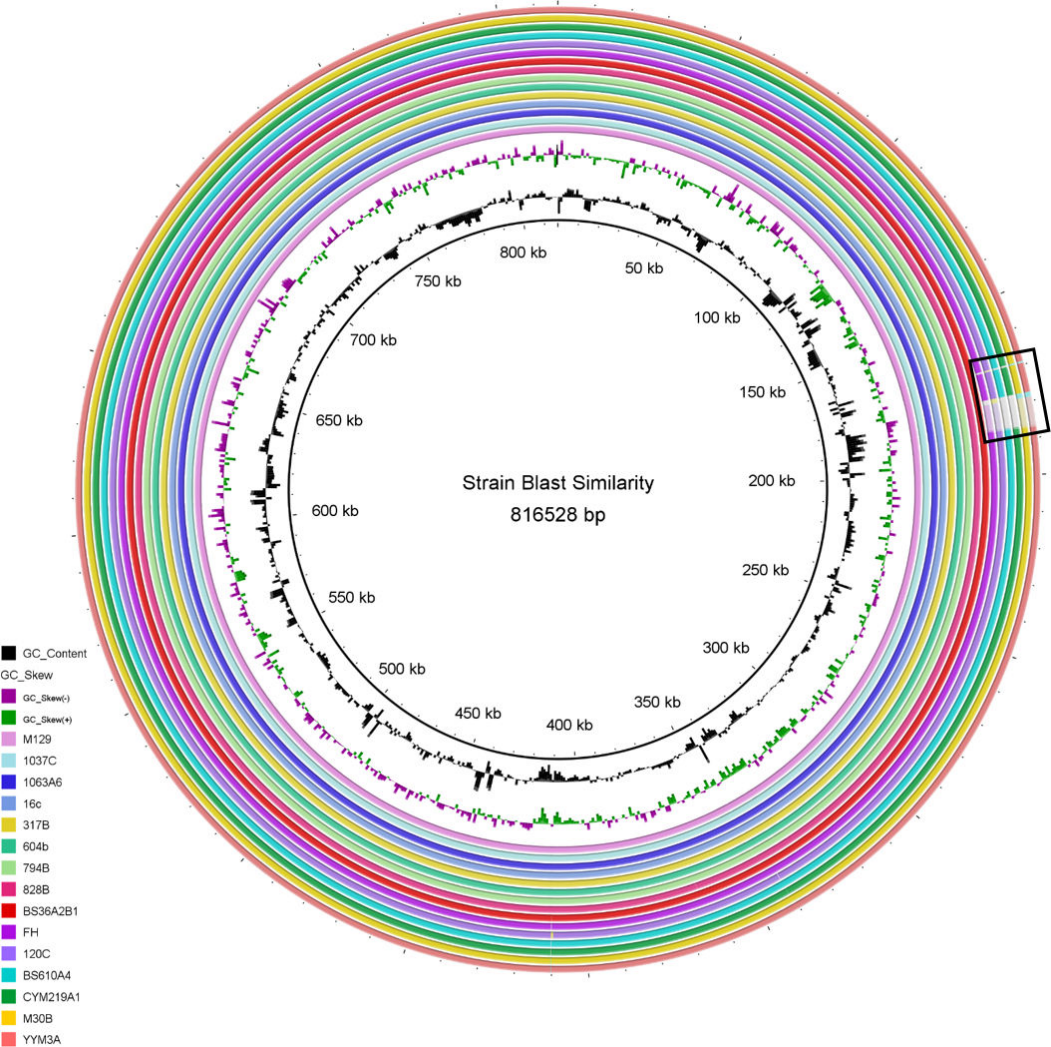
Sequence similarity of the corrected genome. The identity of 14 *M. pneumoniae* strains with the reference strain M129 genome sequence. The similarity of each strain’s genome sequence to the reference genome sequence is represented by a colored circle. The color coding for each strain is provided on the right. The specific position in the reference genome is indicated by the number inside the circle. A solid color indicates a consistency greater than 99%, while a transparent gray color indicates a consistency around 95%.

The genome sequences of the 13 clinical isolates were compared to the M129 reference genome sequence using the BRIG software. It was found that the similarity in most regions of the genome exceeded 99% but decreased to about 95% near the P1 gene in the P1-type II strain (Fig. 1), indicating differences between the P1-type I and P1-type II strains. The disparity between P1-I and P1-II strains is primarily caused by inconsistency in the sequences of the two repeat elements of the P1 genes, repMP4 and repMP2/3.

### Genomic variation

**Fig 2:**
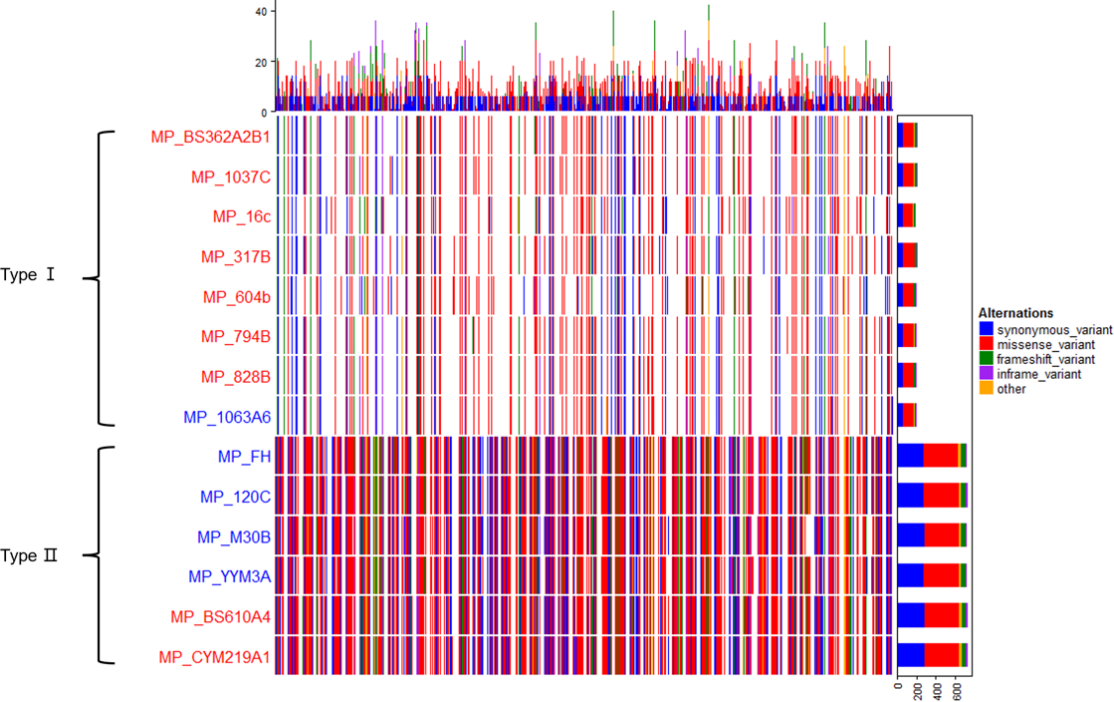
The distribution pattern of variations across distinct sample genomes. Each row in the figure corresponds to all mutation sites of each strain, while each column corresponds to the mutations that occurred in each gene, including synonymous mutations, missense mutations, frameshift mutations, non-frameshift mutations, and other types. Different mutation types are indicated by different colors on the right side of the figure. The strain names on the left side of the figure are marked in red font for resistant strains and in blue font for susceptible strains.

Using M129 as the reference genome, genome-wide variation analysis was conducted on 13 clinical isolates and FH, generating SNP and InDel variants for each strain. The results showed that P1-type II strains exhibited a significantly higher number of mutations compared to P1-type I strains (Table 2). Among SNP variants, missense mutations were the most prevalent, followed by synonymous mutations. Among InDel variants, frameshift mutations were the most common, followed by non-frameshift mutations. The distribution of mutations in the genome was visualized, revealing a clear differentiation between P1-type I and P1-type II strains based on their genotypes. Furthermore, when considering drug resistance, the distribution patterns of mutations in drug-resistant and sensitive strains were generally similar within the same type, with no significant differentiation observed (Fig. 2).

### Phylogenetic tree

**Fig 3:**
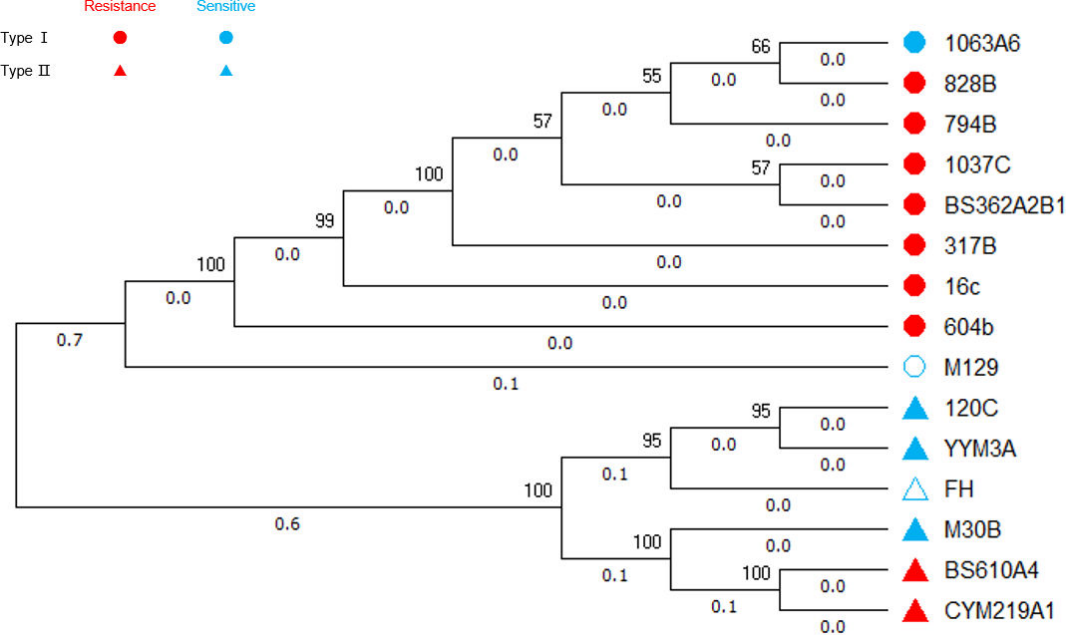
Phylogenetic tree of the strains. The P1-type I branch comprises eight clinical isolates and one reference strain M129, among which only 1063A6 is a sensitive strain; the P1-type II branch comprises five clinical isolates and one reference strain FH, of which three are sensitive strains and two are drug-resistant strains.

The phylogenetic tree, constructed based on the SNP loci, clearly differentiated the 13 clinical isolates, M129, and FH into two distinct branches representing P1-type I and P1-type II strains, respectively. When observing the distribution of drug-resistant and sensitive strains, the phylogenetic tree did not show a distinct separation between them (Fig. 3).

### Drug-resistance gene screening

During the screening of drug-resistant genes in the genome, we observed differences in sequence similarity between *rplD* and *rplV* among different strains. These genes are associated with target modification, and the observed differences were attributed to P1-type rather than resistance. Additionally, we did not identify the *erm* gene responsible for target methylation. In the analysis of sequence similarity of efflux pump-related genes, no differences related to resistance or P1-type were found in the *msrA* and *msrB* genes. Furthermore, the *macB* and *mef* genes were not detected in the genome. Regarding antibiotic inactivation-related genes, no matching genes were found, indicating the absence of antibiotic inactivation-related resistance genes in these strains (Table 3).

**TABLE 3:** Sequence similarity results of resistance genes

Gene	Function	Resistance mechanism	1037C	16c	317B	604b	794B	828B	BS362A2B1	1063A6	120C	M30B	YYM3A	FH	BS610A4	CYM219A1
*rplD*	50S ribosomal protein L4	Target modification	100	100	100	100	100	100	100	100	99.687	99.687	99.687	99.687	99.687	99.687
*rplV*	50S ribosomal protein L22		99.792	99.792	99.792	99.792	99.792	99.792	99.792	99.792	99.583	99.583	99.583	99.583	99.583	99.583
*erm*	23S rRNA [adenine(2058)-N (6)]-methyltransferase		0	0	0	0	0	0	0	0	0	0	0	0	0	0
*msrA*	Peptide-methionine (S)-S-oxide reductase	Antibiotic efflux	99.789	99.789	99.789	99.789	99.789	99.789	99.789	99.789	99.789	99.789	99.789	99.789	99.789	99.789
*msrB*	Peptide-methionine (R)-S-oxide reductase		100	100	99.781	100	100	100	100	100	100	100	99.781	99.781	100	100
*macB*	Macrolide export ATP-binding/permease protein		0	0	0	0	0	0	0	0	0	0	0	0	0	0
*mef*	Macrolide efflux MFS transporter		0	0	0	0	0	0	0	0	0	0	0	0	0	0
*mph*	Macrolide 2'-phosphotransferase	Antibiotic modification	0	0	0	0	0	0	0	0	0	0	0	0	0	0
*mgt*	Macrolide-inactivating glycosyltransferase		0	0	0	0	0	0	0	0	0	0	0	0	0	0
*ere*	Erythromycin esterase		0	0	0	0	0	0	0	0	0	0	0	0	0	0

^*a*^ MFS: major facilitator superfamily.

### Prominent focused variants

**Fig 4:**
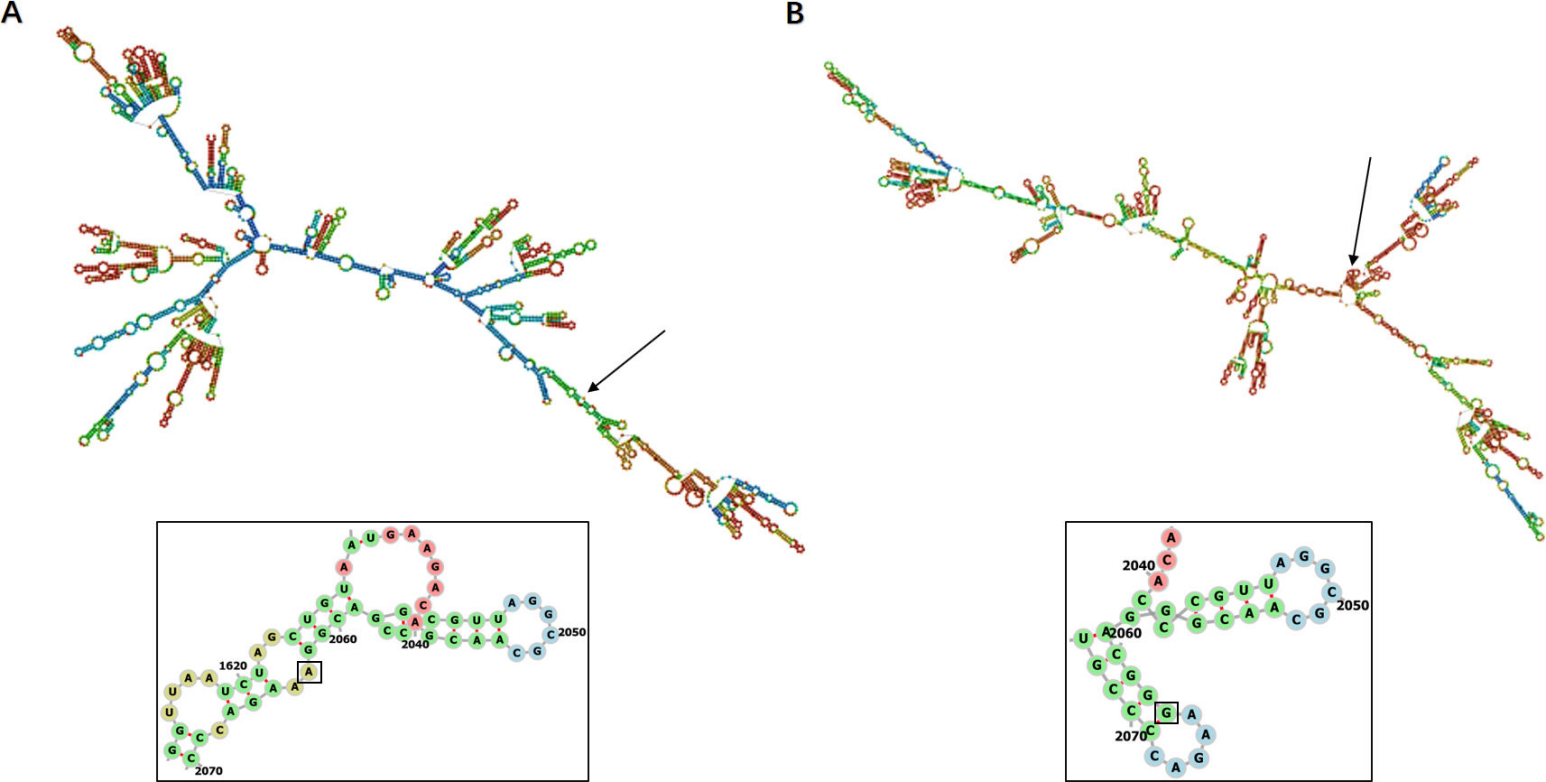
Secondary structure of *M. pneumoniae* 23S rRNA. (**A**) No mutations at position 2063. (**B**) Mutation from A to G at position 2063.

Using M129 as the reference strain, we observed point mutations at positions 2063 and 2064 in the V region of the 23S rRNA (BIX60_RS00545) in 14 strains. Except for the drug-resistant strain 16c, which exhibited a point mutation A2064G, the remaining drug-resistant strains showed a point mutation A2063G in this region, while no mutations were observed in the sensitive strain (Table 4). These mutations were consistent with the amplified sequence of the 23S rRNA gene, and the mutated strains showed high levels of erythromycin resistance (MIC >64 µg/mL) in the *in vitro* susceptibility test, whereas the non-mutated strains remained sensitive (Table 1). Furthermore, no other variants that could clearly distinguish between drug-resistant and susceptible strains were identified. Using the rnafold software (29), we observed that the A2063G mutation of 23S rRNA resulted in a transformation of the stem-loop structure into a complementary base pair at this position, leading to a structural change from a stem loop to a hairpin loop in the RNA secondary structure (Fig. 4). Additionally, an A-AT insertion mutation at position 172 on the 23S rRNA was detected in all P1-type II strains, and it was not associated with drug resistance.

**TABLE 4:** The distribution of focused variants

Position	Annotated gene	Allele change	Protein change	Consequence type	Function	1037C	16c	317B	604b	794B	828B	BS362A2B1	1063A6	BS610A4	CYM219A1	120C	M30B	YYM3A	FH
120202	BIX60_RS00545	A172AT	–	non_coding_transcript _exon_varian	23S ribosomal RNA	–	–	–	–	–	–	–	–	√	√	√	√	√	√
122093	BIX60_RS00545	A2063G	–	non_coding_transcript _exon_variant	23S ribosomal RNA	√	–	√	√	√	√	√	–	√	√	–	–	–	–
122094	BIX60_RS00545	A2064G	–	non_coding_transcript _exon_variant	23S ribosomal RNA	–	√	–	–	–	–	–	–	–	–	–	–	–	–
218958	BIX60_RS00975	C162A	Thr54Thr	synonymous _variant	50S ribosomal protein L4	–	–	–	–	–	–	–	–	√	√	√	√	√	√
219226	BIX60_RS00975	A430G	Met144Val	missense _variant	50S ribosomal protein L4	–	–	–	–	–	–	–	–	√	√	√	√	√	√
221482	BIX60_RS00995	T204C	Ala68Ala	synonymous _variant	50S ribosomal protein L22	–	–	–	–	–	–	–	–	√	√	√	√	√	√
221711	BIX60_RS00995	T433C	Ser145Pro	missense _variant	50S ribosomal protein L22	√	√	√	√	√	√	√	√	√	√	√	√	√	√
445585	BIX60_RS02140	T1112G	Ile371Ser	missense _variant	ADP-ribosylating toxin CARDS	–	–	–	–	–	–	–	–	√	√	√	√	√	√
33450	BIX60_RS00155	C394T	Arg132Cys	missense _variant	Glycosyl transferase	–	–	–	√	–	–	–	–	–	–	–	–	–	–
507617	BIX60_RS02445	G1137A	Ala379Ala	synonymous _variant	MFS transporter	–	√	–	–	–	–	–	–	–	–	–	–	–	–
85726	BIX60_RS00395	C700A	Gln234Lys	missense _variant	rRNA(cytidine-2′-O-)-methyltransferase	–	–	–	√	–	–	–	–	–	–	–	–	–	–
140380	BIX60_RS00635	CA363C	Val122fs	frameshift _variant	Adenine-specific methyltransferase	–	√	–	–	–	–	–	–	–	–	–	–	–	–

^*a*^ A checkmark (√) indicates the presence of the mutation in the corresponding strain of the column, while a dash (–) indicates its absence.

Furthermore, we observed the distribution of mutations in the 50S ribosomal proteins L4 (BIX60_RS00975) and L22 (BIX60_RS00995). Two point mutations occurred in the L4 gene: a synonymous mutation C162A and a missense mutation A430G, both of which occurred only in P1-type II strains. Similarly, the L22 gene exhibited two point mutations: a synonymous mutation T204C and a missense mutation T433C, with the T204C mutation also specific to P1-type II strains (Table 4). However, these four point mutations did not show a clear distribution pattern among drug-resistant and sensitive strains. In addition to the previously mentioned mutations, we identified additional mutations in specific genes. The 604B-resistant strain had a missense mutation C394T in the gene BIX60_RS00155, which is annotated as a glycosyltransferase involved in antibiotic inactivation in the macrolide resistance mechanism. Furthermore, the 604B strain also had a missense mutation C700A in the gene BIX60_RS00395, while the 16C strain exhibited a deletion mutation CA363C in the gene BIX60_RS00635. These two genes are annotated as methyltransferases, similar to the methyltransferase function performed by the erm gene. However, these mutations were not observed in other resistant strains. The 16C strain, a clinically isolated P1-resistant strain in 2003, was associated with a case of *Streptococcus pneumoniae* meningitis in a child who also presented with pleural effusion and abnormal liver function. On the other hand, the 604B strain, a P1-resistant strain collected clinically in 2008, was associated with a case of common pneumonia in a child, without any specific intra- or extra-pulmonary complications. The significance of these two strains and their mutations requires further investigation for confirmation.

The CARDS gene (BIX60_RS02140), an important virulence factor of *M. pneumoniae*, exhibited a missense mutation T1112G that was found in all P1-type II strains, consistent with previously reported results (30), and appeared to be more associated with the P1-type.

### Strain-specific gene analysis

The comparative genome analysis of the three-generation sequencing strains revealed specific genomic phenomena in strains 16C, 120C, YYM3A, BS610A4, and CYM219A1 (Table S3). Each group represents a homologous cluster, providing a list of sequences belonging to this cluster in different samples. In Group658, strain 16C was annotated as a pseudogene, likely due to a CA-C frame-shift deletion at position 140380 in the genome. Group658 functions as an adenine-specific DNA methyltransferase, similar to the methyltransferase function performed by the erm gene. Further investigation is needed to determine the association between the loss of this gene function and macrolide drug resistance. In Group663, strain 120C was annotated as a pseudogene, possibly resulting from a TA-T shift mutation at position 715600 in the genome. The function of Group663 was annotated as a DUF31 family protein. In Group664 and Group668, strains BS610A4 and CYM219A1 exhibited loss of gene function (MPN647 lipoprotein and PfpI family protein) due to the same frameshift mutation. These two strains were classified as P1-II type resistant strains and were associated with clinical presentations of *S. pneumoniae* meningitis with pleural effusion. Treatment with macrolide antibiotics alone was ineffective, necessitating the addition of glucocorticoid therapy and bronchial lavage after admission. In Group669, Group670, and Group671, strains 120C and YYM3A also exhibited loss of gene function (P80 lipoprotein, cell division, and ABC transporter) due to the same frameshift mutation. Both strains belonged to the P1-II type sensitive strains and clinically presented as common MPP without any complications.

Additionally, we observed gene multicopy phenomena in strains BS610A4 and CYM219A1 (Fig. S3), with the fragment being 480 bp in length and annotated as a pseudoprotein. When searching for this fragment in the blast database, we found gene duplications present in seven published *M. pneumoniae* genomes (Table S4). Currently, based on the distribution of this fragment in the strains, no direct correlation with drug resistance or P1 type has been identified. Further analysis of the functional domains in this segment revealed that it shares a conserved functional domain, Petidase_S7 superfamily, with the DUF31 protein family, suggesting a similar function. The BS610A4 and CYM219A1 isolates were clinically diagnosed with SMPP.

## DISCUSSION

In this study, we conducted a comprehensive analysis of 13 clinical isolates collected and cultured between 2003 and 2019, including P1 typing, detection of drug-resistance mutations, *in vitro* drug sensitivity testing, whole-genome sequencing, comparative genomics, and analysis of clinical information. The drug-resistant strains all exhibited point mutations in the V region of the 23S rRNA, with A2063G being the predominant mutation site, resulting in a high level of resistance to erythromycin. Comparative genomics analysis revealed that the difference in P1 type among *M. pneumoniae* strains was significantly greater than the difference between macrolide antibiotic-resistant and susceptible strains. The genome sequences of P1-I type and P1-II type strains exhibited high consistency, with the main variations concentrated in the P1 gene region. The drug resistance analysis revealed a strong correlation between the A2063G point mutation in 23S rRNA and macrolide resistance. All strains causing severe pneumonia were drug-resistant, but further exploration is needed to identify the specific gene changes in their genomes.

The genome of *M. pneumoniae* is highly streamlined, and since the release of the genome information, it has been resequenced and annotated by Xiao et al. using third-generation sequencing technology (30). Complete genome sequences have also been reported by several countries. The genome of *M. pneumoniae* is highly conserved, with less than 1% variation among strains, primarily in the repeat sequence of the P1 protein, which serves as a major immune and adhesion structure. Based on the characteristics of repeated sequences, *M. pneumoniae* can be divided into two types, distinguished mainly by the Rep2/3 and Rep4 fragments. By analyzing the genome data of *M. pneumoniae*, we observed significant differences between P1-type I and P1-type II strains in terms of the distinct distribution trend of variation sites, as well as the effective classification of strains into two branches by the constructed phylogenetic tree. However, the distribution pattern of variation sites in P1-I type drug-resistant and sensitive strains exhibited a high level of consistency, consistent with the findings of Kenri et al. in their molecular epidemiological study of *M. pneumoniae* isolates in Japan from 2006 to 2019, where they also failed to differentiate between drug-resistant and sensitive strains (31). Alterations in the P1 gene can lead to structural changes in the P1 protein, affecting its interaction with host respiratory epithelial cells and film-forming properties, which may be associated with pathogenicity (32). The difference in the prevalence of severe pneumonia between P1-type I and P1-type II in this study was not statistically significant, and there was also no difference in the combined complications among severe cases. The analysis conducted by Fan et al. on the correlation between *M. pneumoniae* type and clinical manifestations indicated that P1-type I strains were more likely to lead to severe cases (33). However, the findings of Nilsson et al. demonstrated that the clinical manifestations following *M. pneumoniae* infection were independent of the type and instead depended on the pathogenic viral load (2, 34). Therefore, the higher infection rate of the P1-I strain can be attributed to its dominance in certain regions. In addition to the P1 gene, our analysis of specific genes revealed certain point mutations that were exclusively present in P1-type II strains. For instance, in the L4 gene, we observed the C162A and A430G mutations exclusively in all P1-type II strains, which agree with the findings reported by Zhao et al. in 2019. However, we found a slight discrepancy in the T204C mutation in the L22 gene compared to their report of the T279C mutation (9). Additionally, in the CARDS gene, missense mutations T1112G were detected in all P1-type II strains, consistent with the results reported by Xiao et al. (30). These mutations could potentially serve as rapid markers for P1 typing and be applied in clinical surveillance.

Point mutations in the V region of 23S rRNA lead to changes in RNA secondary structure (Fig. 4), enabling the ineffective attachment of macrolide molecules to ribosomal peptidyltransferases, leading to drug resistance in *M. pneumoniae* (35). The internationally reported mutation positions in 23S rRNA are 2063, 2064, and 2617, with the A2063G mutation being the most common in China, followed by the A2064G mutation, which accounts for over 90% of clinically resistant strains (7, 36). In this study, we performed genome-wide variation analysis on all strains and identified that strain 16c harbored the A2064G point mutation, while the remaining drug-resistant strains carried the A2063G point mutation. Conversely, no mutations at these two positions were observed in all sensitive strains. These findings were consistent with the results of drug susceptibility testing, suggesting that this specific locus could effectively distinguish between drug-resistant and sensitive strains. Apart from this locus, no other variants with distinguishable patterns between drug-resistant and sensitive strains were identified. Mutations in L4 and L22 have been implicated in macrolide resistance in other species (37), but in this study, no gene mutations directly associated with drug resistance were identified, which aligns with the findings reported by Zhao et al. in 2019 (9). We also analyzed other reported resistance genes associated with macrolide resistance mechanisms, including erm (target modification), mef, msr, macB (drug efflux mechanism), and various hydrolases and inactivation enzymes. However, these resistance genes were not detected in our *M. pneumoniae* isolates, and the presence of these resistance mechanisms in *M. pneumoniae* needs to be further investigated. Macrolide antibiotics are considered the first-line treatment for *M. pneumoniae* infections in children, and infections caused by macrolide-resistant strains of *M. pneumoniae* (38) are closely associated with clinical outcomes. Since the first report of macrolide-resistant clinical isolates of *M. pneumoniae* in Japan in 2001, numerous domestic and international clinical studies have consistently shown that children with macrolide-resistant *M. pneumoniae* (MRMP) infections experience a longer duration of fever and hospital stay compared to those infected with macrolide-sensitive *M. pneumoniae*. Timely replacement of sensitive antibiotics can effectively improve the clinical course in MRMP infections (7, 36). It is important to note that infection with drug-resistant strains of *M. pneumoniae* is strongly associated with the development of severe disease. All severe cases in this project were infected with drug-resistant strains.

The pathogenesis of *M. pneumoniae* is complex, involving various factors such as the host and strain. Apart from direct tissue damage, *M. pneumoniae* can induce abnormal immune responses, leading to the development of chronic infections and airway inflammatory responses, indicating that immune evasion may play a role. The MIB-MIP system, containing *Mycoplasma* Ig binding protein (MIB) and *Mycoplasma* Ig binding protease (MIP), is widespread in Mollicutes. It is responsible for the capture and cleavage of IgG and acts as an effective factor for evading the host immune system (39). DUF 31 is regarded as an MIP-like protein, playing an important role in evading host Ig-mediated defenses (40). In this study, the strain-specific analysis revealed that BS610A4 and CYM219A1 had multiple copies of a gene (Fig. S3). Further investigation revealed that this gene shares the conserved domain Petidase_S7 superfamily with the DUF31 protein family, indicating its potential similarity in functions to DUF31, a serine protease domain associated with the pathogenicity of *M. pneumoniae* (39). BS610A4 is a clinical isolate from 2019 presenting with SMPP, accompanied by pleural effusion, abnormal liver function, and myocardial damage. It belongs to P1-type II and carries an A2063G point mutation in region V of 23S rRNA. Clinical treatment included glucocorticoid therapy and two bronchoalveolar lavages, with visible sputum plugs in the lavage fluid. CYM219A1, isolated in 2016, also presented with SMPP and pleural effusion. It belongs to P1-type II and carries the A2063G point mutation in region V of 23S rRNA. Clinical treatment consisted of glucocorticoid therapy and bronchoalveolar lavage. BS362A2B1 displayed clinical manifestations similar to these two strains but lacked this specific functional gene alteration. Further confirmation is required to establish its relevance to the clinical scenario.

In this study, clinical isolates of *M. pneumoniae* from different ages and types were selected for P1 typing, drug resistance detection, and whole-genome sequencing. These analyses were combined with clinical information for comprehensive analysis, aiming to explore possible clinical correlations. The main limitation of this study is the small number of clinical cases, and the specific genetic alterations that need to be verified require an expansion of the clinical sample size. In strain P1-type II, we identified three specific gene mutations: C162A and A430G mutations in the L4 gene and T1112G mutation in the CARDS gene, which could potentially serve as a rapid typing method for *M. pneumoniae*. BS610A4 and CYM219A1 display the same gene duplication phenomenon. Although the gene is functionally annotated as a pseudoprotein, it shares a conserved functional domain with the DUF31 protein family. Clinically, both cases present with severe RMPP requiring glucocorticoid therapy and are accompanied by pleural effusion. These findings suggest a potential association with the clinical manifestations and warrant further exploration. Although no specific genes were directly associated with macrolide antibiotic resistance or clinical severity, comparisons revealed specific genetic alterations between different types and strains. Further investigation with an expanded sample size and comprehensive analytical approach, combining comparative genomics and clinical manifestations, is necessary to provide theoretical support for exploring the relationship between pathogenic mechanisms, strain characteristics, and clinical manifestations of *M. pneumoniae*.

## Data Availability

The raw sequence data reported in this paper have been deposited in the Genome Sequence Archive (41) in National Genomics Data Center (42), China National Center for Bioinformation/Beijing Institute of Genomics, Chinese Academy of Sciences (GSA: CRA011769), and are publicly accessible at https://ngdc.cncb.ac.cn/gsa. The whole-genome sequence data reported in this paper have been deposited in the Genome Warehouse (https://ngdc.cncb.ac.cn/gwh) (43) and GenBase (https://ngdc.cncb.ac.cn/genbase) (44). Detailed accession numbers can be found in Table S5.

## SUPPLEMENTAL MATERIAL

Supplemental material spectrum.03615-23-s0001.docx Tables S1 to S5; Fig. S1 to S3.

ASM does not own the copyrights to Supplemental Material that may be linked to, or accessed through, an article. The authors have granted ASM a non-exclusive, world-wide license to publish the Supplemental Material files. Please contact the corresponding author directly for reuse.
