# Supplementary material for: Comparative genomic sequencing to characterize Mycoplasma pneumoniae genome, typing, and drug resistance
Source: Microbiol Spectr. 2024 Jun 21;12(8):e03615-23. doi: 10.1128/spectrum.03615-23 (PMC11302288; doi:10.1128/spectrum.03615-23)
Supplement: Supplemental material — Tables S1 to S5; Fig. S1 to S3. [file spectrum.03615-23-s0001.docx]

**Supplementary Table 1 The sequences of the PCR primers for the experiment**

|  | Primer name | Primer sequence（5′→3′） | Product length(bp) |
| --- | --- | --- | --- |
| 16S rRNA | 16sF | TCT GAG AGT TTG ATC CTG GCT | 1520 |
|  | 16sR | CCA TTA GAA AGG AGG TGA TCC A |  |
| 23S rRNA domain II | IIF | GTT ACT AAG GGC TTA TGG TGG ATG | 816 |
|  | IIR | CCA AGG CAT CCA CCA TAA G |  |
| 23S rRNA domain V | LP3 | TAA CTA TAA CGG TCC TAA GG | 853 |
|  | LP4 | CAC ACT TAG ATG CTT TCA GCG |  |


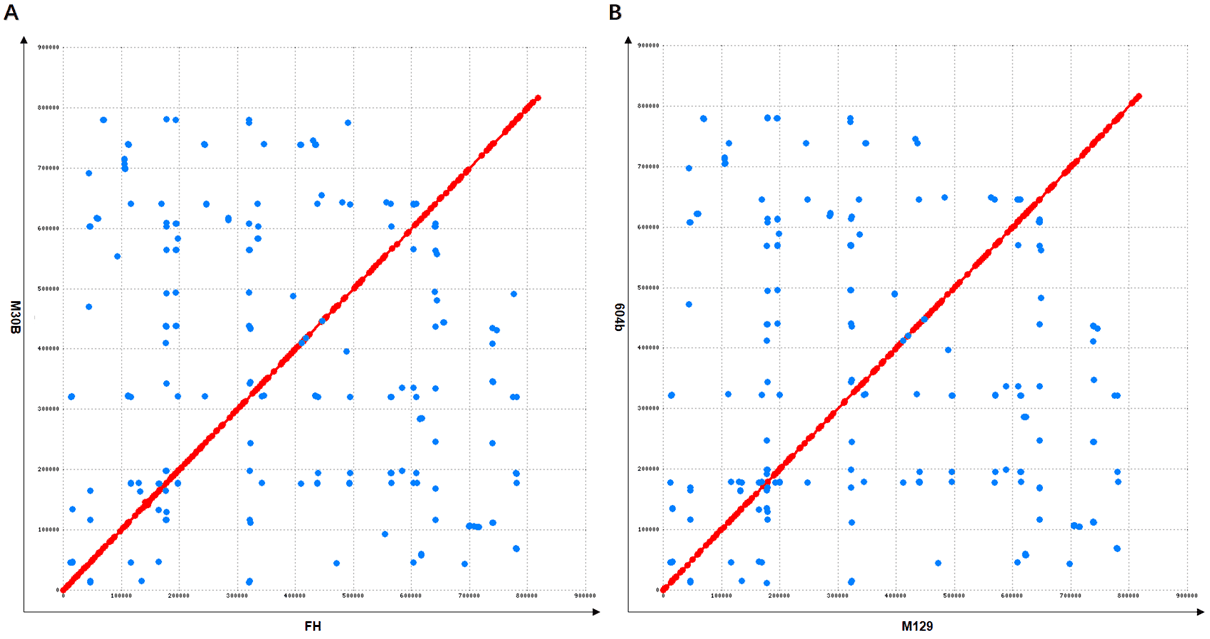


**Supplementary Figure 1 Alignment of corrected assembly results with the reference genome**

Note: (A) Alignment of the corrected assembly results of the P1-type II strain M30B with the reference genome FH; (B) Alignment of the corrected assembly results of the P1-type I strain 604b with the reference genome M129.

**Supplementary Table 2 Statistics of the assembly results of the second-generation data corrected by the reference genome**

| Strain | MR mutations | P1typing | Reference genome | Total original splice length（bp） | Total length after correction（bp） | GC（%） |
| --- | --- | --- | --- | --- | --- | --- |
| 16c | R | Ⅰ | M129 | 783,231 | 813,523 | 40.00 |
| 120C | S | Ⅱ | FH | 805,787 | 816,566 | 39.98 |
| 317B | R | Ⅰ | M129 | 805,872 | 816,140 | 40.00 |
| 604b | R | Ⅰ | M129 | 762,940 | 816,276 | 40.00 |
| 794B | R | Ⅰ | M129 | 816,716 | 816,066 | 40.00 |
| 828B | R | Ⅰ | M129 | 798,367 | 816,297 | 40.00 |
| 1037C | R | Ⅰ | M129 | 825,599 | 817,081 | 39.96 |
| 1063A6 | R | Ⅰ | M129 | 808,368 | 816,045 | 40.00 |
| M30B | S | Ⅱ | FH | 815,427 | 816,801 | 39.97 |
| YYM3A | S | Ⅱ | FH | 810,038 | 814,874 | 39.99 |

Note: S=drug-sensitive strain, R=drug resistant strain;


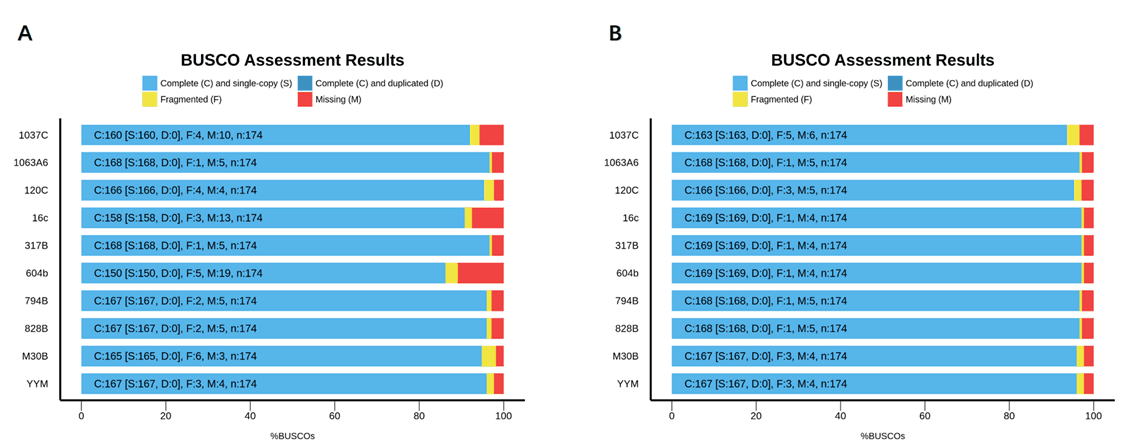


**Supplementary Figure 2 Comparison of gene completeness of genome assembly results in the second generation**

（A）Soapdenovo assembly results；（B）Corrected using the reference genome after Soapdenovo assembled

**Supplementary Table 3 Specific genes found in the genomes of different strains**

| Group | Group function | 1037C | 1063A6 | 120C | 16C | 317B | 794B | BS362A2B1 | BS610A4 | CYM219A1 | YYM | Mutations | Consequence |
| --- | --- | --- | --- | --- | --- | --- | --- | --- | --- | --- | --- | --- | --- |
| Group658 | class I SAM-dependent methyltransferase | 1037C_000125 | 1063A6_000125 | 120C_000126 | **16c_000124*** | 317B_000125 | 794B_000125 | BS362A2B1_000124 | BS610A4_000128 | CYM219A1_000128 | YYM_000126 | 140380 CA-C | Frameshift |
| Group663 | DUF31 family protein | 1037C_000670 | 1063A6_000670 | **120C_000668*** | 16c_000668 | 317B_000669 | 794B_000672 | BS362A2B1_000670 | BS610A4_000672 | CYM219A1_000672 | YYM_000669 | 715600 TA-T | Frameshift |
| Group664 | MPN647 family lipoprotein | 1037C_000716 | 1063A6_000716 | 120C_000716 | 16c_000714 | 317B_000715 | 794B_000718 | BS362A2B1_000716 | **BS610A4_000720*** | **CYM219A1_000720*** | YYM_000717 | 765931 AGCATCTTTTT-A | Frameshift |
| Group668 | DJ-1/PfpI family protein | 1037C_000034 | 1063A6_000034 | 120C_000034 | 16c_000034 | 317B_000034 | 794B_000034 | BS362A2B1_000034 | **BS610A4_000034*** | **CYM219A1_000034*** | YYM_000034 | 35988 A-AT | Frameshift |
| Group669 | P80 family lipoprotein | 1037C_000322 | 1063A6_000323 | **120C_000317*** | 16c_000319 | 317B_000321 | 794B_000323 | BS362A2B1_000322 | BS610A4_000320 | CYM219A1_000320 | **YYM_000318*** | 346291 G-GA | Frameshift |
| Group670 | cell division protein FtsZ | 1037C_000359 | 1063A6_000360 | **120C_000354*** | 16c_000356 | 317B_000358 | 794B_000360 | BS362A2B1_000359 | BS610A4_000357 | CYM219A1_000357 | **YYM_000355*** | 376887 T-TA | Frameshift |
| Group671 | ABC transporter permease | 1037C_000375 | 1063A6_000376 | **120C_000370*** | 16c_000372 | 317B_000374 | 794B_000376 | BS362A2B1_000375 | BS610A4_000373 | CYM219A1_000373 | **YYM_000371*** | 395001 GA-G | Frameshift |

*: The gene name in bold indicates that it has been annotated as a pseudogene; YYM=YYM3A


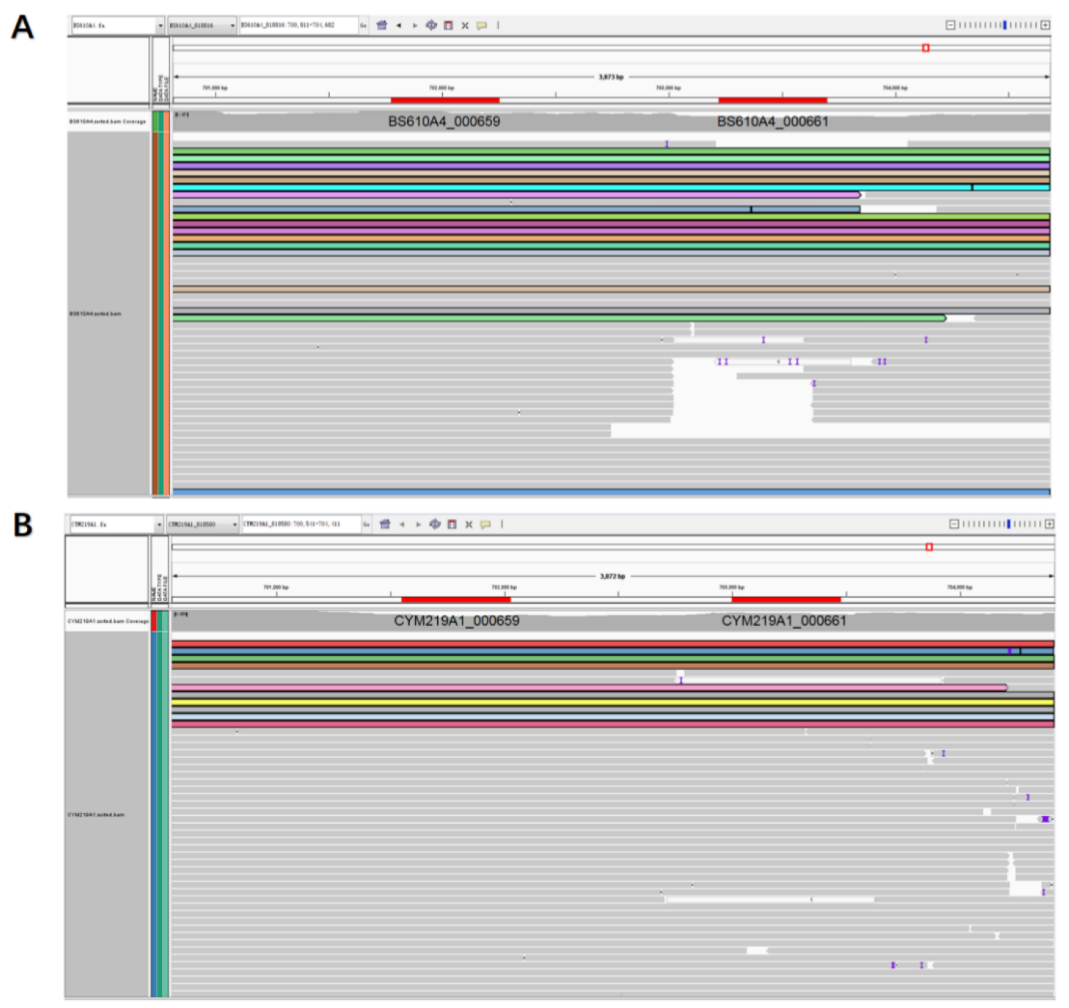


**Supplementary Figure 3 Multiple-copy gene present in the BS610A4 strain and CYM219A1 strain**

Note: (A) Multi-copy phenomenon in strain BS610A4: gene BS610A4_000661 is a multi-copy gene of gene BS610A4_000659, and IGV visualization shows multiple reads spanning this interval; (B) Multi-copy phenomenon in strain CYM219A1: gene CYM219A1_000661 is a multi-copy gene of gene CYM219A1_000659, and IGV visualization shows multiple reads spanning this interval

**Supplementary Table 4 Statistics of the gene copy numbers in each genome**

| Stain |  | MR | Copy number | Accession | Origin | Year |
| --- | --- | --- | --- | --- | --- | --- |
| Type I | 16c | R | 1 | GWHDOMW01000000 | China | 2003 |
|  | 317B | R | 1 | GWHDONA01000000 | China | 2006 |
|  | 794B | R | 1 | GWHDOMY01000000 | China | 2009 |
|  | 1037C | R | 1 | GWHDONB01000000 | China | 2010 |
|  | 1063A6 | S | 1 | GWHDONC01000000 | China | 2010 |
|  | BS362A2B1 | S | 1 | GWHDONE01000000 | China | 2017 |
|  | M129 | S | 1 | CP017343.1 | - | - |
| Type II | 120C | S | 1 | GWHDOMZ01000000 | China | 2005 |
|  | YYM3A | S | 1 | GWHDOMX01000000 | China | - |
|  | FH | S | 1 | CP017327.1 | - | - |
|  | 1006 | R | 1 | CP017337.1 | USA | 1999 |
|  | CYM219A1 | R | 2 | GWHDOND01000000 | China | 2016 |
|  | BS610A4 | R | 2 | GWHDONF01000000 | China | 2019 |
|  | 16-734 | S | 2 | CP039761.1 | Korea | 2016 |
|  | 14-637 | S | 2 | CP039772.1 | Korea | 2014 |
|  | 11-1384 | S | 2 | CP039775.1 | Korea | 2011 |
|  | 10-1385 | S | 2 | CP039784.1 | Korea | 2010 |
|  | CO3 | S | 2 | CP017342.1 | USA | 2014 |
|  | 519 | S | 2 | CP017339.1 | USA | 1995 |
|  | E57 | S | 2 | CP017329.1 | Egypt | 2009 |

**Supplementary Table 5 The accession numbers for the genome sequences of 13 Mycoplasma pneumoniae strains.**

| Strain | Level | GWH Accession | GenBase Accession |
| --- | --- | --- | --- |
| 16c | Chromosome | GWHDOMW01000000 | C_AA062336.1 |
| YYM3A | Chromosome | GWHDOMX01000000 | C_AA062345.1 |
| 794B | Chromosome | GWHDOMY01000000 | C_AA062341.1 |
| 120C | Chromosome | GWHDOMZ01000000 | C_AA062338.1 |
| 317B | Chromosome | GWHDONA01000000 | C_AA062340.1 |
| 1037C | Chromosome | GWHDONB01000000 | C_AA062336.1 |
| 1063A6 | Chromosome | GWHDONC01000000 | C_AA062337.1 |
| CYM219A1 | Chromosome | GWHDOND01000000 | C_AA062344.1 |
| BS362A2B1 | Chromosome | GWHDONE01000000 | C_AA062342.1 |
| BS610A4 | Chromosome | GWHDONF01000000 | C_AA062343.1 |
| M30B | Scaffold | GWHDONG00000000 | C_AA062586.1-C_AA062772.1 |
| 604b | Scaffold | GWHDONH00000000 | C_AA062496.1-C_AA062585.1 |
| 828B | Scaffold | GWHDONI00000000 | C_AA062346.1-C_AA062495.1 |
